# Assessment of the Accuracy of Determining the Angular Position of the Unmanned Bathymetric Surveying Vehicle Based on the Sea Horizon Image

**DOI:** 10.3390/s19214644

**Published:** 2019-10-25

**Authors:** Krzysztof Naus, Łukasz Marchel, Piotr Szymak, Aleksander Nowak

**Affiliations:** 1 Faculty of Navigation and Naval Weapons, Polish Naval Academy, Smidowicza 69, 81–103 Gdynia, Poland; l.marchel@amw.gdynia.pl; 2 Faculty of Mechanical and Electrical Engineering, Polish Naval Academy, Smidowicza 69, 81–103 Gdynia, Poland; p.szymak@amw.gdynia.pl; 3 Faculty of Civil and Environmental Engineering, Department of Geodesy, Gdansk University of Technology, Narutowicza 11/12, 80–233 Gdansk, Poland; aleksander.nowak@pg.edu.pl

**Keywords:** bathymetric surveying, roll and pitch, image of the sea horizon

## Abstract

The paper presents the results of research on assessing the accuracy of angular position measurement relative to the sea horizon using a camera mounted on an unmanned bathymetric surveying vehicle of the Unmanned Surface Vehicle (USV) or Unmanned Aerial Vehicle (UAV) type. The first part of the article presents the essence of the problem. The rules of taking the angular position of the vehicle into account in bathymetric surveys and the general concept of the two-camera tilt compensator were described. The second part presents a mathematical description of the meters characterizing a resolution and a mean error of measurements, made on the base of the horizon line image, recorded with an optical system with a Complementary Metal-Oxide Semiconductor (CMOS) matrix. The phenomenon of the horizon line curvature in the image projected onto the matrix that appears with the increase of the camera height has been characterized. The third part contains an example of a detailed analysis of selected cameras mounted on UAVs manufactured by DJI, carried out using the proposed meters. The obtained results including measurement resolutions of a single-pixel and mean errors of the horizon line slope measurement were presented in the form of many tables and charts with extensive comments. The final part presents the general conclusions from the performed research and a proposal of directions for their further development.

## 1. Introduction

An important factor affecting the accuracy of bathymetric surveys performed with sonar, echosounder or Light Detection and Ranging (LiDAR) is the lack of correlation between a location of the positioning system antenna mounted on the surveying vehicle (surface type, e.g., USV, or air type, e.g., UAV) and a reflection point of the sound or light wave (depending on the type of the sensor) on the seabed [1]. Under ideal weather and propagation conditions, the acoustic or light ray is a straight line with a constant direction relative to the vertical. It allows to link the measured depth with the location of the positioning system antenna using constant values of coordinate rotation parameters which transform the coordinate system of the measuring sensor to the coordinate system connected with the Earth, most often WGS-84 [2].

However, in real conditions, due to sea waves and wind, it is necessary to take the pitch and roll of the surveying vehicle into account in each epoch of bathymetric measurement. Only on their basis, it is possible to determine the variable direction shifts of acoustic or light rays from a vertical line and then the coordinates (e.g., WGS 84) of the point of the sound/light wave reflection from the seabed [3].The problem can be solved by applying appropriate methods of the pitch and roll compensation. Currently, most of them use the information concerning spatial orientation angles obtained from Inertial Navigation Systems (INSs) which use for pitch and roll determination Global Navigation Satellite System (GNSS) receivers and Inertial Measurement Units (IMUs) of the Microelectromechanical Systems (MEMS) type. An IMU comprises tri–axial accelerometers and gyroscopes and is typically coupled with a magnetic flux sensor. Sensor fusion of the IMU readings allow measuring 3D orientation with respect to a fixed system of coordinates. Therefore, when an IMU is firmly attached to a UAV body, it is possible to obtain an estimate of its absolute orientation [4]. However, MEMS IMU outputs are corrupted by significant sensor errors. The navigation errors of a MEMS-based INS will therefore accumulate very quickly over time. This requires aiding from other sensors such as Global Navigation Satellite Systems (GNSS) [5]. IMU errors can be classified into two types: deterministic errors and random errors. Major deterministic error sources including constant bias, scale factor errors and misalignment. They can be removed by calibration and compensation. The random constant bias (turn to turn bias) and random noises are the main error sources in the orientation-finding system [6,7]. In the case of light surface bathymetric surveying vehicles of the USV type, the following INSs can be found [8]:

- Ekinox determining roll & pitch with RMS = 0.05° (RTK outage–30 s),
- Apogee determining roll & pitch z RMS = 0.012° (RTK outage–60 s),
- Horizon determining roll & pitch z RMS = 0.01° (RTK outage–60 s).

While, in the case of light aerial bathymetric surveying vehicles of the UAV type, the following INS series are used:

- Trimble Direct Mapping Solution (DMS) for example mounted on UAV RIEGL’s BathyCopter–determining roll & pitch with RMS in range of (0.015°, 0.2°) [9,10],
- Ellipse 2 cooperating with ASTRALiTe’s edge LiDAR mounted on UAV DJI Matrice 600 Pro UAV determining roll & pitch with RMS = 0.1° [11,12].

However, having in mind the rapid development of sea bathymetry, aimed at making more accurate measurements using UAV and USV autonomously [13,14,15], it can be expected to look for new methods of determining the spatial orientation angles.

**Figure 1:**
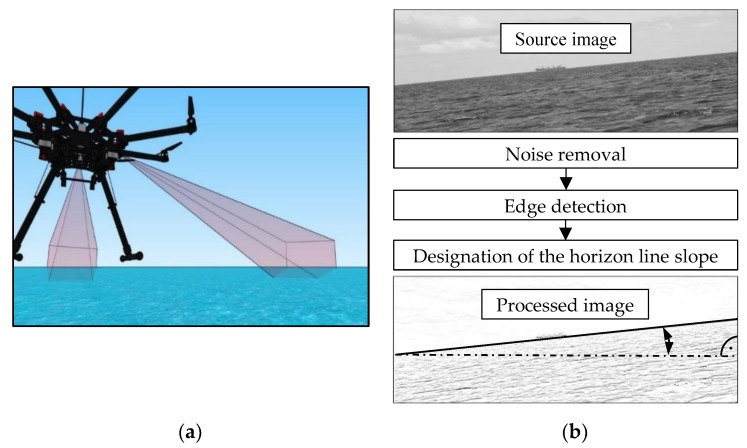
The idea of measuring the horizon line slope: (**a**) Recording images with a two-camera tilt compensator mounted on a UAV; (**b**) The image processing process for the slope angle.

As a proposal for an interesting and perspective solution, one can indicate a method based on observing the horizon line slope in the camera image [16,17]. This type of tilt compensator could be built from two cameras recording images (mounted perpendicular to each other on a vehicle with optical axes directed to the horizon) and a microcomputer that processes them into roll and pitch angles–measured as angles between the horizontal edge of the image and the extracted line of the horizon (Figure 1).

Due to the fact many effective methods of detecting the horizon line in the image have been developed [18,19] and a very wide range of light, high-resolution cameras are available, it seems advisable to determine with what accuracy can be measured the angular position of UAV and USV using a horizon line observation by the camera?

It should be additionally noted that the literature lacks information on this subject. The information may be very important when making decisions about taking further research and development works (R&D), raising the technology readiness level for new solutions based on the image of the sea horizon. They may include not only those used in bathymetric surveying but e.g., for stabilizing the spatial orientation of the flight, positioning of objects floating on the sea using UAVs, matching of the seabed 3D imaging maps for comparative purposes, etc.

Therefore, this article attempts to describe the method of assessing the measuring accuracy of the horizon line slope using a camera. It is supplemented with an example of the possibility of its practical application for the precision analysis of selected cameras mounted on USV manufactured by DJI [20].

## 2. Methods

**Figure 2:**
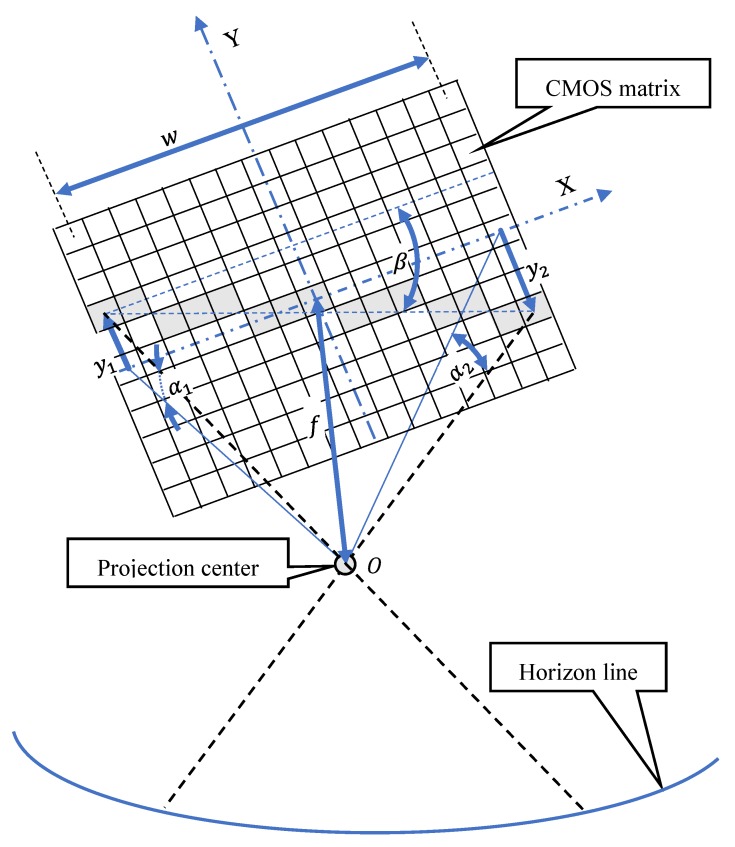
The idea of measuring the horizon line slope with a CMOS matrix camera.

Figure 2 presents the idea of measuring the horizon line slope with a CMOS matrix camera.

Accuracy of measuring the horizon line slope using a CMOS (Complementary Metal-Oxide Semiconductor) matrix camera can be characterized by two parameters: mean error and resolution. Their sizes depend mainly on the focusing of the optical system and the pixel size on the matrix. Focusing possibilities determine the “focusing power” of light rays. The shorter the focal length is, the stronger the lens refracts the rays. It means that focuses them more, causing moving the image away and reducing the measurement accuracy. The typical size of a single-pixel in modern CMOS sensors is from 1.7 to 14 micrometers. The smaller pixel size ensures better reproduction of image details and thus increases measurement accuracy.

The mean error of the horizon line slope measurement β can be determined by applying the law of mean errors propagation formulated by C. F. Gauss. Knowing the mean errors of independent variables f, w, $\alpha_{1}$ and $\alpha_{2}$ of a single measurement result function:

(1) $$\beta =arc\;\tan\frac{\sqrt{w^{2}+4\cdot f^{2}}\cdot \left(\tan\alpha_{1}+\tan\alpha_{2}\right)}{2\cdot w},$$

the mean error equation can be easily written:

(2) mβ=[(∂β∂f·mf)2+(∂β∂w·mw)2+(∂β∂α1·mα1)2+(∂β∂α2·mα2)2]12,

which after determining the partial derivatives will take the form:

(3) mβ=[(2·f·(tanα1+tanα2)w·w2+4·f2·((w2+4·f2)·(tanα1+tanα2)24·w2+1)·mf)2+(tanα1+tanα22·w2+4·f2−w2+4·f2·(tanα1+tanα2)2·w2(w2+4·f2)·(tanα1+tanα2)24·w2+1·mw)2+(w2+4·f2·(tan2α1+1)2·w·((w2+4·f2)·(tanα1+tanα2)24·w2+1)·mα1)2+(w2+4·f2·(tan2α2+1)2·w·((w2+4·f2)·(tanα1+tanα2)24·w2+1)·mα2)2]12,

where:

$m_{f}$—mean error of focal length measurement,
$m_{w}$—mean error of CMOS matrix width measurement,
$m_{\alpha_{1}},\;m_{\alpha_{2}}$—mean error of angles $\alpha_{1}$ i $\alpha_{2}$ measurements on CMOS matrix.

**Figure 3:**
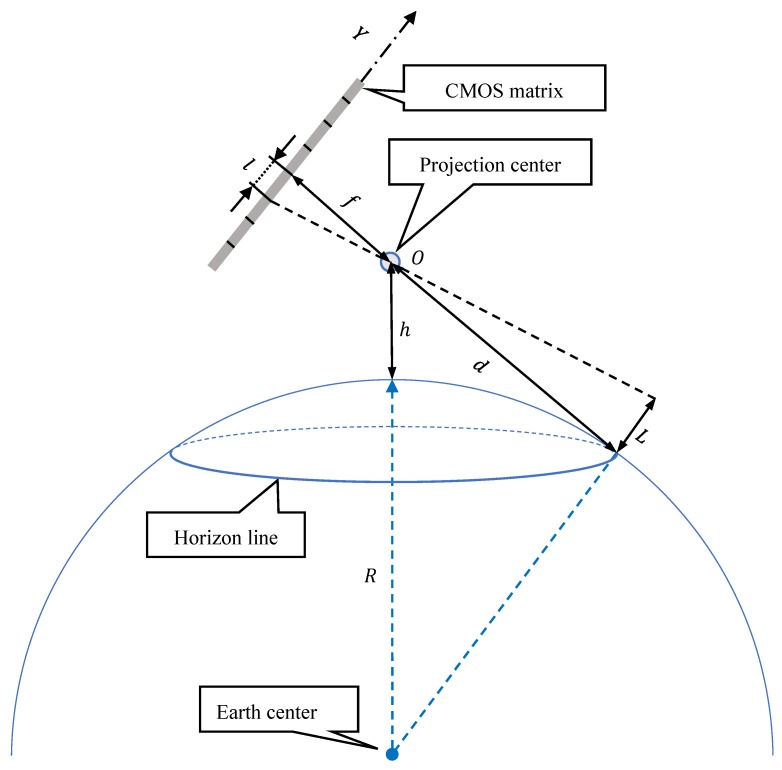
Graphic interpretation of the measurement resolution of a single-pixel representing the horizon line on the matrix.

While, using the similarity of right-angled triangles (Figure 3), the formula for the measurement resolution of a single-pixel L representing the horizon line on the matrix, can be easily derived. It will take the following form:

(4) $$L=\frac{l\cdot d}{f},$$

where:

l—pixel size (calculated as the ratio of matrix height in units of length to matrix height in pixels),
f—focal length,
d—distance to the horizon line.

Where in [21,22]:

(5) $$d=\sqrt{2\cdot R\cdot h},$$

or taking the phenomenon of light refraction in the Earth’s atmosphere into account:

(6) $$d=\frac{\sqrt{2\cdot R\cdot h}\;}{\sqrt{1-k}},$$

where:

R—length of the Earth’s radius,
h—camera height above sea level (a.s.l.),
k—Earth’s refraction coefficient (depending on the state of the atmosphere: pressure, temperature and humidity) [21,22,23,24,25].

Then, based on the known measurement resolution of single-pixel and horizontal field of view of the camera ($FOV_{H}$):

(7) $$FOV_{H}=2\cdot \mathrm{atan}\frac{w}{2\cdot f},$$

the measurement resolution of the horizon line slope can be calculated:

(8) $$r_{\beta }=\mathrm{atan}\frac{L}{z}=\frac{l\cdot \sqrt{1+\frac{w^{2}}{4\cdot f^{2}}}}{w},$$

where:

(9) $$z=2\cdot d\cdot \sin\frac{FOV_{H}}{2}.$$

**Figure 4:**
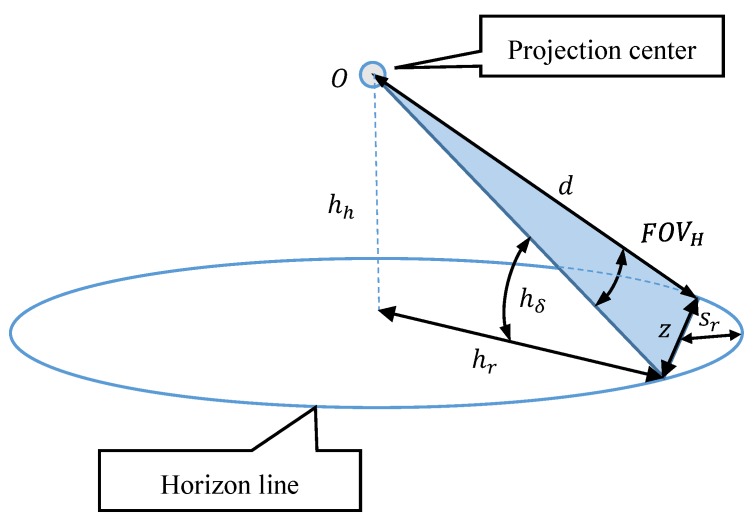
Part of the horizon line (with the length z) in the $FOV_{H}$ of the camera.

In Equation (7) it was assumed that the part of the horizon line (with the length z) in the horizontal field of view of the camera ($FOV_{H}$) has the shape of a line, not an arc (Figure 4).

**Figure 5:**
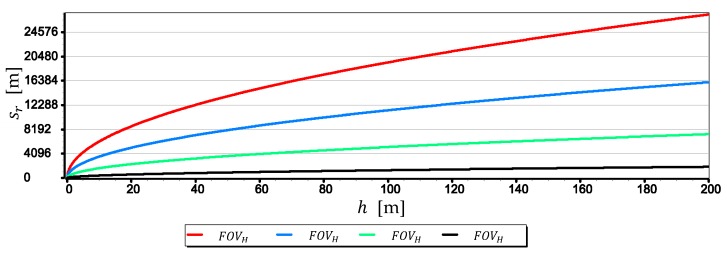
Horizon sagitta $s_{r}$ as a function of the height of the camera a.s.l. h (for $FOV_{H}$ equals 30°, 60°, 90° and 120°, *R* = 6,378,000.0 m, *k* = 0.16).

It was decided to simplify it because (as already shown in Figure 2) only the extreme pixels of the matrix (which are also boundary $FOV_{H}$) are used to calculate the horizon line slope β. However, it should be realized that even for low AUV flight altitudes, but large $FOV_{H}$ values of horizon sagitta $s_{r}$ can be significant. Figure 5 presents the graphs of the horizon sagitta $s_{r}$ for several selected $FOV_{H}$ as a function of the height of the camera a.s.l. h. Calculation were performed using the following relation:

(10) $$s_{r}=h_{r}\cdot \left(1-\cos\frac{FOV_{H}}{2}\right)\;,$$

where radius of horizon circle:

(11) $$h_{r}=\frac{d\cdot R}{h+R}.$$

**Figure 6:**
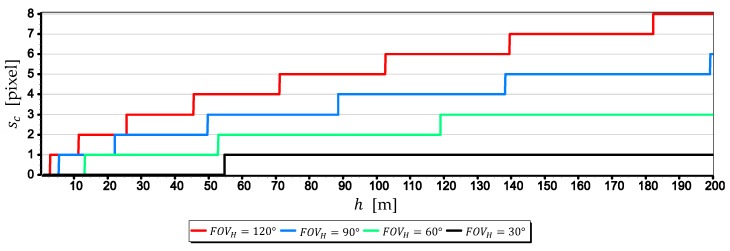
Horizon sagitta $s_{c}$ as a function of the height of the camera a.s.l. h (for $FOV_{H}$ equals 30°, 60°, 90° and 120°, R = 6,378,000.0 m, k = 0.16, $p_{H}$ = 4000 pixels).

While, Figure 6 shows the horizon sagitta $s_{c}$ as the number of pixels on the matrix, calculated using the following formula:

(12) $$s_{c}=\frac{p_{H}\cdot h_{h}\cdot \cos h_{\delta }\;-\;h_{r}\cdot \cos\frac{FOV_{H}}{2}\cdot \sin h_{\delta }}{2\cdot h_{r}\cdot \sin\frac{FOV_{H}}{2}},$$

where:

(13) $$h_{h}=R-\sqrt{R^{2}-h_{r}{}^{2}}+h,$$

(14) $$h_{\delta }=arc\;tan\frac{h_{h}}{h_{r}},$$

$p_{H}$—horizontal resolution of the matrix in pixels.

In Calculation (12) it was assumed that the optical axis of the camera is directed at a point lying on the horizon line, as shown in Figure 2.

The charts presented in Figure 5 testify the high values of the horizon sagitta $s_{r}$. But these values do not translate directly into the shape of the horizon line seen in the image. Due to the large linear distortions of the image that occur at small angles between the camera’s optical axis line and the horizon plane, this arc will always be larger than the real one (“straightened”). This is clearly seen in Figure 6, where the $s_{c}$ value for $FOV_{H}=30{}^{\circ}$ and h=200 m corresponds to just one pixel on the matrix, and for $FOV_{H}=120{}^{\circ}$ and h=200 m–eight pixels.

## 3. Research and Discussion

Four different cameras mounted on UAVs manufactured by DJI were evaluated. They were chosen primarily because they differ in optical parameters, including focal lengths as well as matrix sizes and resolutions. Their most important technical parameters taken in the calculations into account are presented in Table 1.

The measures of assessment were the measurement resolution and the mean error of measurement calculated using the formulas presented in Section 2, for:

- R = 6,378,000.0 m,
- k = 0.16 (average value of refraction coefficient for the Baltic Sea),
- h = (0 m, 200 m),
- f = 4 mm (DJI FC350), 5 mm (DJI FC220), (4 mm, 14 mmm) (DJI FC550RAW), 35 mm (SONY ILCE-7RM2);

And:

(13) $$m_{w}=w/n_{p},$$

(14) $$m_{\alpha_{1}}=m_{\alpha_{2}}=arc\tan\left(l/\sqrt{w^{2}+f^{2}}\right),$$

where: $n_{p}$—number of pixels in a row of the matrix, $m_{f}=l$.

### 3.1. Measurement Resolution Analysis

**Figure 7:**
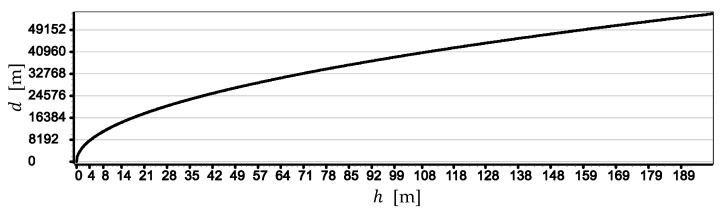
Distance to the horizon line d as a function of the height of the camera a.s.l h.

For a better bringing of the problem of moving the horizon line away from the camera, Figure 7 presents a graph of distance to the horizon line d as a function of the height of the camera a.s.l. h.

The graph in Figure 7 clearly shows that when the camera reaches a height of 10 m, the distance to the horizon line is already 12,000 m and with a further increase in camera height it increases linearly up to 55,000 m.

#### 3.1.1. Measurement Resolution of a Single-Pixel

**Figure 8:**
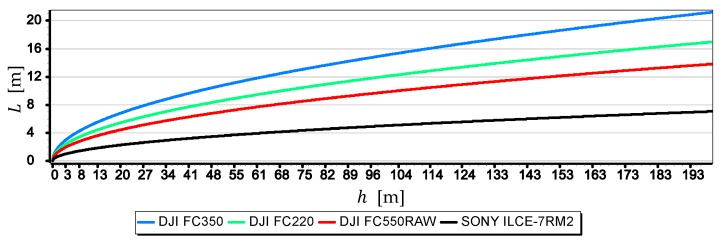
Single-pixel measurements resolution L of the horizon line as a function of the height of the camera a.s.l. h.

Figure 8 presents the graphs of a single-pixel measurements resolution L of the horizon line taken with cameras: DJI FC350, DJI FC220, DJI FC550RAW and SONY ILCE-7RM2.

**Figure 9:**
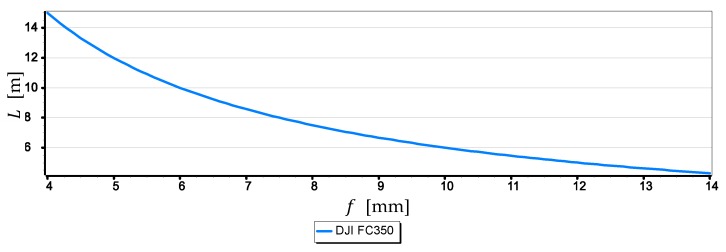
Single-pixel measurement resolution L of the horizon line as a function of focal length f from 4 mm to 14 mm for camera DJI FC5350 (h = 100 m).

Graphs in Figure 8 show that the measurement resolutions of a single-pixel L decrease significantly after reaching by cameras a height of 8 m a.s.l., although they are still in a quite narrow range (1.5 m, 4 m). What cannot be said after the cameras reach a height of 200 m a.s.l., when this range is wide (7 m, 21 m)–it should be remembered that the distance to the horizon d is then up to 55,000 m. The graphs allow also to rank the cameras from the best to the worst, assessing them in terms of obtained the single-pixel measurement resolution L, in the following order: SONY ILCE-7, DJI FC550RAW, DJI FC220, DJI RM2FC350. Figure 9 shows the graphs of the single-pixel measurement resolution L of the horizon line as a function of focal length f from 4 mm to 14 mm for camera DJI FC5350.

**Figure 10:**
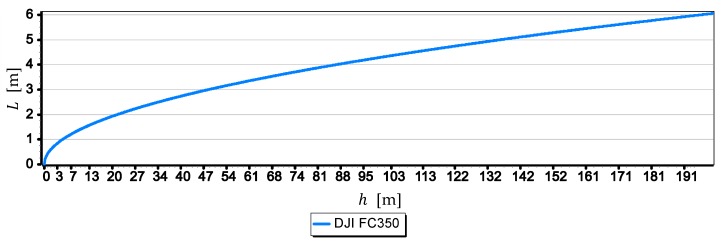
Single-pixel measurements resolution L of the horizon line as a function of the height of the DJI FC350 camera a.s.l. h (f = 14 mm).

The graph in Figure 9 shows that to obtain a higher single-pixel measurement resolution of the horizon line, the focal length should be increased. Therefore, the focal length measurement resolution of 4 mm for the DJI FC350 camera (see Figure 8) taken to the previous calculations was incorrect and resulted in the lowest measurement resolution. Thus, to show the essence of the indicated problem, Figure 10 presents a graph of the single-pixel measurement resolution L of the horizon line taken with a DJI FC350 camera, but at a maximum focal length setting of f = 14 mm.

Figure 10 shows a significant improvement in the single-pixel measurement resolution L of the horizon line. In the case of a measurement made at an altitude of 200 m a.s.l. the resolution increased from 21 m to 6 m. However, it should be remembered that by increasing the focal length f we reduce the horizontal field of view $FOV_{H}$. For FC350 camera with matrix width w = 6.16 mm, $FOV_{H}$ decreases from 75.2° (for f = 4 mm) to 24.8° (for f = 14 mm).

#### 3.1.2. Resolution of the Horizon Slope Measurement

To make the comparison of the rated cameras with each other easier, Table 2 presents their horizon slope measurement resolution $r_{\beta }$ and horizontal field of view $FOV_{H}$ calculated using Equations (7) and (8).

**Figure 11:**
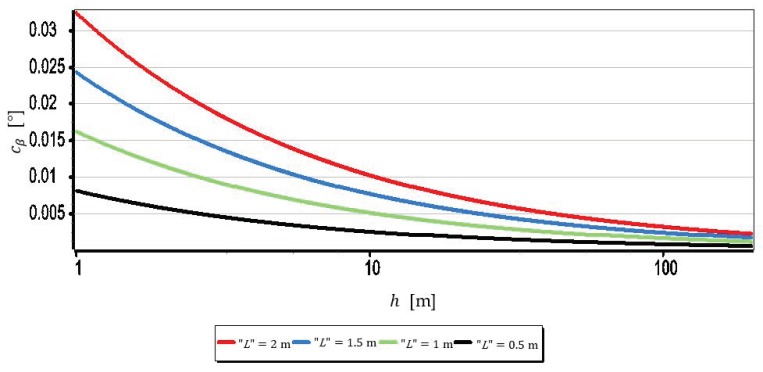
Measurement resolution of the horizon slope $c_{\beta }$ as a function of h for chosen wave heights “L” ($FOV_{H}$ = 53.91°–SONY ILCE-7RM2).

**Figure 12:**
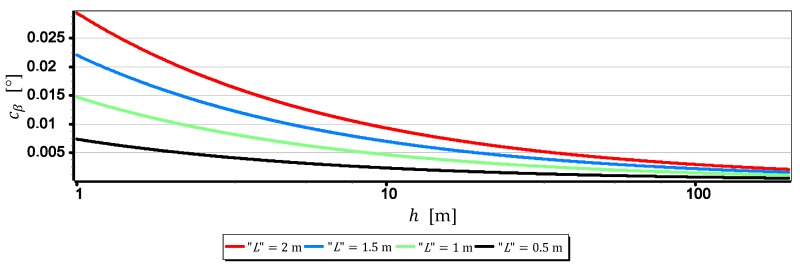
Measurement resolution of the horizon slope $c_{\beta }$ as a function of h for chosen wave heights “L” ($FOV_{H}$ = 59.94°–DJI FC550RAW).

**Figure 13:**
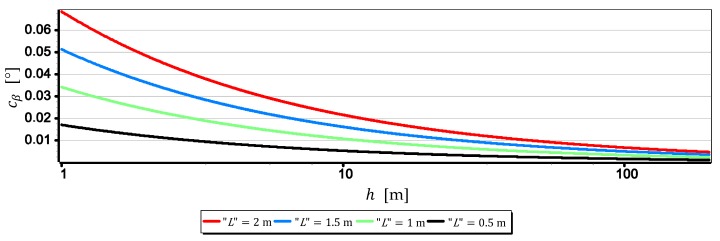
Measurement resolution of the horizon slop $c_{\beta }$ as a function of h for chosen wave heights “L” ($FOV_{H}$ = 24.81°–DJI FC350).

**Figure 14:**
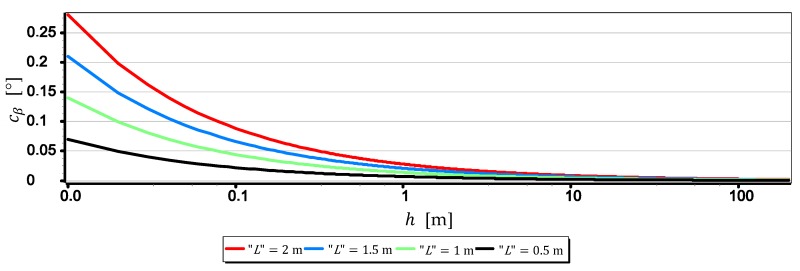
Measurement resolution of the horizon slop $c_{\beta }$ as a function of h a.s.l. for chosen wave heights “L” ($FOV_{H}$ = 63.27°–DJI FC220).

The results set out in Table 2 show that the measurements of the horizon slope β can be performed with a resolution of 0.02°. Nevertheless, it should be remembered that the horizon is almost always distorted at the contact with the sea by rippling the water surface. Therefore, increasing the single-pixel measurement resolution L to values smaller than the height of the sea waves certainly will not increase the measurement resolution of the horizon slop $r_{\beta }$. Therefore, it is reasonable to check at which values of $FOV_{H}$ and h, sea waving should be included in the computations. The dependence (9) can be easily used for this purpose. It should only be assumed that the L value corresponds to the height of the sea waves-further referred to as “L”. The result of calculations obtained at that time can be treated as the horizon slope measurement resolution–further referred to as $c_{\beta }$, depending on $FOV_{H}$ and the wave height “L”. Figure 11, Figure 12, Figure 13 and Figure 14 presents $c_{\beta }$ charts for four arbitrarily selected wave heights “L”, equal: 0.5 m, 1 m, 1.5 m and 2 m (corresponding to sea states 2–4 of the Douglas Sea Scale [26]).

When comparing the $r_{\beta }$ values in Table 2 with the $c_{\beta }$ values shown in Figure 11, Figure 12, Figure 13 and Figure 14, it can be stated that $c_{\beta }$ may be greater than $r_{\beta }$. Therefore, when talking about the actual measurement resolution of the horizon slop using a camera, $c_{\beta }$ for small h and $r_{\beta }$ for large h should be used. Table 3 presents the threshold values of height $h_{c_{\beta }/r_{\beta }}$ above which $r_{\beta }$ should be used instead of $c_{\beta }$.

**Figure 15:**
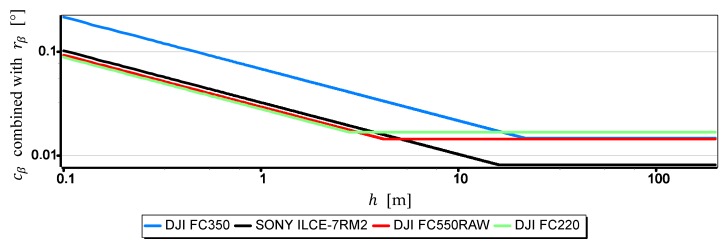
Real measurement resolution of the horizon slope for the wave height “L” = 2 m.

On the other hand, Figure 15 presents graphs of the real measurement resolution of the horizon slope for the wave heights “L” = 2 m. They arose as a result of combining a part of the diagram $c_{\beta }$ below $h_{c_{\beta }/r_{\beta }}$ with a part of the diagram $r_{\beta }$ above $h_{c_{\beta }/r_{\beta }}$.

The introduction of $c_{\beta }$ resolution in the range $(0,\;h_{c_{\beta }/r_{\beta }}]$ and $r_{\beta }$ in the range $\left(h_{c_{\beta }/r_{\beta }},\;+\infty \right)$ gives the possibility of a better comparative assessment of the cameras used to measure β. For example, based on Table 3 and Figure 15, it can be unequivocally stated that the measurement resolution β taken at low altitudes (below 3.0 m) with DJI FC550RAW and DJI FC220 cameras is similar to the resolution of the SONY ILCE-7RM2 camera–something completely different show data in Table 2. Another example is, that the constant (and also the largest) measurement resolution β (equal to $r_{\beta }$) can be obtained with a DJI FC550RAW camera from a height of h = 4.2 m, and with a DJI FC350 camera only after reaching a height of h = 21.7 m.

### 3.2. Analysis of the Mean Error of Measurement

**Figure 16:**
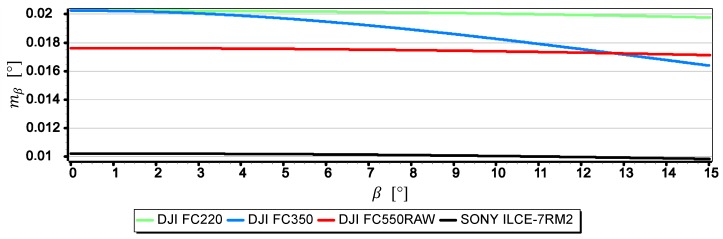
Mean error of measurement $m_{\beta }$ as a function of the horizon slope β.

For the calculation of the mean error $m_{\beta }$ of the horizon slope β measurement made with the assessed cameras, the method described in Section 2 was used–Equation (3). The results obtained in this way are presented in Figure 16 in the form of four diagrams of the mean error $m_{\beta }$ of the horizon slope β measurement for values in the range (0°,15°).

**Figure 17:**
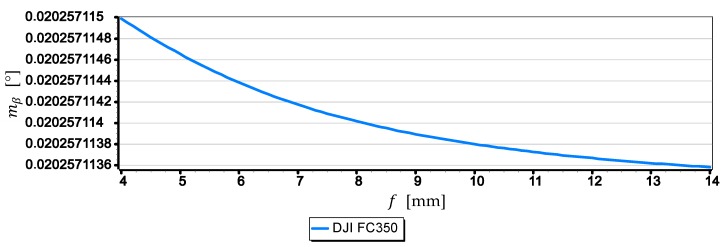
Mean error of measurement $m_{\beta }$ as a function of focal length f of the DJI FC350 camera (β=0°).

**Figure 18:**
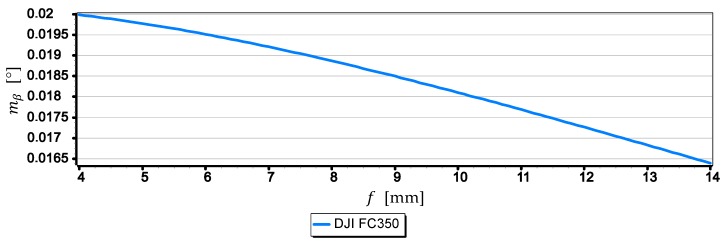
Mean error of measurement $m_{\beta }$ as a function of focal length f of the DJI FC350 camera (β=15°).

The graphs presented in Figure 16 show that the smallest mean error (not exceeding 0.012°) is for measurements taken with the SONY ILCE-7RM2 camera, while the largest (0.021°) for measurements taken with the DJI FC220 camera. Compared with other graphs, the diagram for the DJI FC350 camera deserves special attention, as it can be clearly seen that the mean error decreases the most with the increase of the horizon slope β. To explain this phenomenon, Figure 17 and Figure 18 present graphs of the mean error of measurement $m_{\beta }$ over the full range of focal length of the DJI FC350 camera (4 mm ≤f ≤14 mm) for β=0° and β=15°.

**Figure 19:**
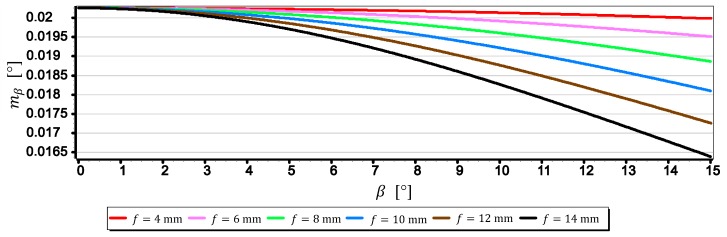
Mean error of measurement $m_{\beta }$ for DJI FC350 camera as a function of the horizon slope β=(0°,15°) for focal length f: 4 mm, 6 mm, 8 mm, 10 mm, 12 mm and 14 mm.

The graph in Figure 17 shows that the focal length f has no major impact on the value of the mean error of measurement $m_{\beta }$ for close to zero values of horizon slope (β≈0°). However, the graph in Figure 18 shows that for β=15°, an increase in focal length from 4 mm to 14 mm will reduce the value of the mean error $m_{\beta }$ by about 25%. To better show this phenomenon, Figure 19 presents diagrams of the mean error of measurement $m_{\beta }$ taken with the DJI FC350 camera as a function of the horizon slope β=(0°,15°) at a focal length f: 4 mm, 6 mm, 8 mm, 10 mm, 12 mm and 14 mm (chosen arbitrarily).

Based on previous considerations and analysis of the diagrams presented in Figure 19, a generalized conclusion can be drawn that in order to measure any value of the horizon slope β with the smallest mean error ($m_{\beta }=min$), the focal length should be set on a maximum value (f=max) and the camera should be rotated relative to the optical axis so that the horizon line would be along the diagonal of the matrix.

#### 3.2.1. Analysis of the Impact of $m_{f},m_{w},m_{\alpha_{1}},m_{\alpha_{2}}$ on $m_{\beta }$

The mean measurement errors $m_{f},m_{w},m_{\alpha_{1}},m_{\alpha_{2}}$ of selected elements (parameters) of the camera’s optical system were analyzed, taken into account in the calculation of the mean measurement error $m_{\beta }$. Nevertheless, for a better understanding of their meaning, they were considered separately. The results of calculations of the single component value from the Formula (3) were used. They correspond to the mean measurement error of a particular element of the camera optical system (e.g., focal length, matrix size). For example, for the $m_{f}$ analysis, the $m_{\beta }$. value was calculated using only the first component, and the remaining three were completely omitted.

#### 3.2.2. Analysis of $m_{f}$

**Figure 20:**
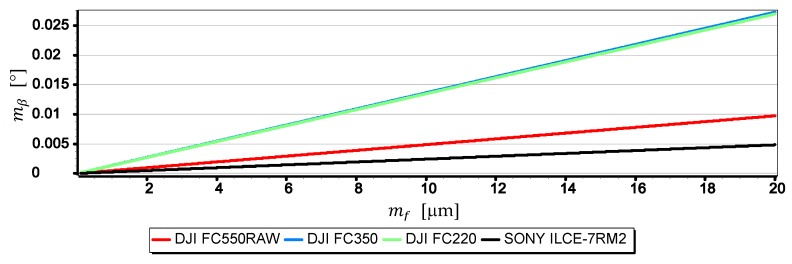
Mean error of measurement $m_{\beta }$ as a function of $m_{f}$ for β=5° (only the first component of the Formula (3) was used in the calculations).

Figure 20 presents the graph of the mean error of measurement error $m_{\beta }$ as a function of the focal length mean measurement error for the horizon slop angle *β* = 5° (a description of the methods and accuracy of focal length measurement can be found, among others in [27,28,29,30]).

Then, Table 4 presents the mean error of measurement $m_{\beta }$ calculated for β=5° and β=10° assuming that $m_{f}=10$ μm.

Based on the graphs presented in Figure 20, a generalized conclusion can be formulated that the accuracy of measuring the focal length $m_{f}$ has a large impact on the value of $m_{\beta }$. In the case of DJI FC550RAW, DJI FC350 for $m_{f}=20$ μm the value $m_{\beta }$ increases significantly reaching 0.027°. However, the results of calculations presented in Table 4 for $m_{f}=10$ μm, β=10° and β=15° indicate that the value of $m_{\beta }$ increases slightly as a function of the angle *β*. In the case of the SONY ILCE-7RM2 camera, the $m_{\beta }$ value increased by only 0.0013°. In the case of SONY ILCE-7RM2 camera, the value of $m_{\beta }$ increased by only 0.0013°.

#### 3.2.3. Analysis of $m_{w}$

**Figure 21:**
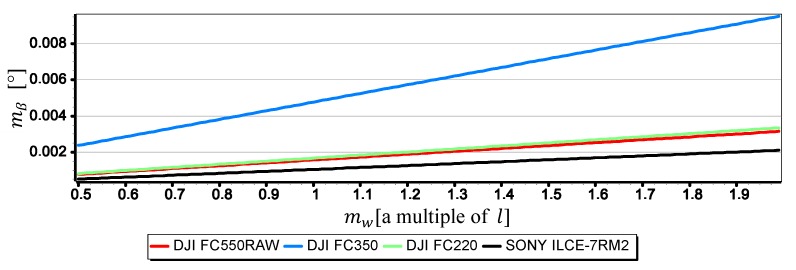
Mean error of measurement $m_{\beta }$ as a function of $m_{w}$ for β=5° (only the second component of the Formula (3) was used in the calculations).

**Figure 22:**
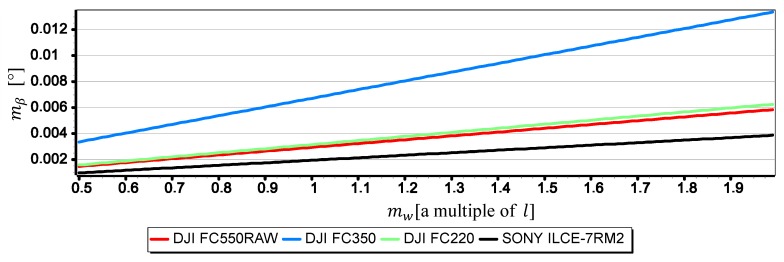
Mean error of measurement $m_{\beta }$ as a function of $m_{w}$ for β=10° (only the second component of the Formula (3) was used in the calculations).

**Figure 23:**
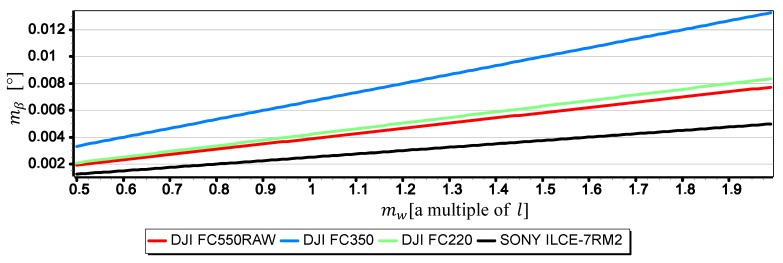
Mean error of measurement $m_{\beta }$ as a function of $m_{w}$ for β=15° (only the second component of the Formula (3) was used in the calculations).

Figure 21, Figure 22 and Figure 23 present graphs of the mean error of measurement $m_{\beta }$ as a function of mean error of CMOS matrix width measurement $m_{w}$ calculated for the horizon slope β equal 5°, 10°, 15°. It was assumed that the value of $m_{w}$ corresponds to a multiple of l in the range of (0.5l,2l)—where l corresponds to pixel size calculated as the ratio of matrix height in units of length to matrix height in pixels (like in Section 2). It made possible to carry out an analysis of the impact of $m_{w}$ on $m_{\beta }$ using the comparative method for all the tested cameras simultaneously, even though they have COMS matrices of different sizes and resolutions.

The graphs presented in Figure 21, Figure 22 and Figure 23 show that the impact of $m_{w}$ on $m_{\beta }$ depends to a large extent on the size of the angle β being measured. With the increase of β from 5° to 15°, the value of $m_{\beta }$ increases up to about 2.5 times. However, this value is still not very big-in the case of DJI FC220, DJI FC550RAW, SONY ILCE-7RM2 cameras it remains in the ranges of thousandths of a degree.

#### 3.2.4. Analysis of $m_{\alpha_{1}},m_{\alpha_{2}}$

**Figure 24:**
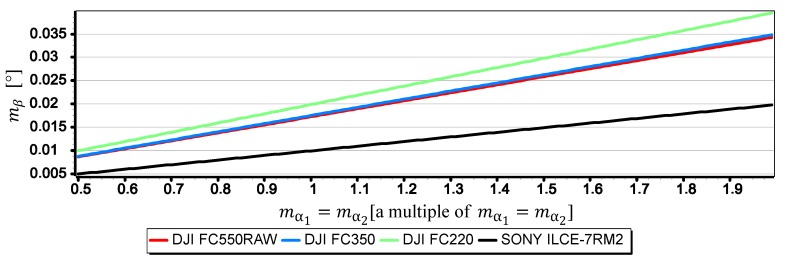
Mean error of measurement $m_{\beta }$ as a function of $m_{\alpha_{1}}=m_{\alpha_{2}}$ for β=5° (only the last two components of the Formula (3) were used in the calculations).

**Figure 25:**
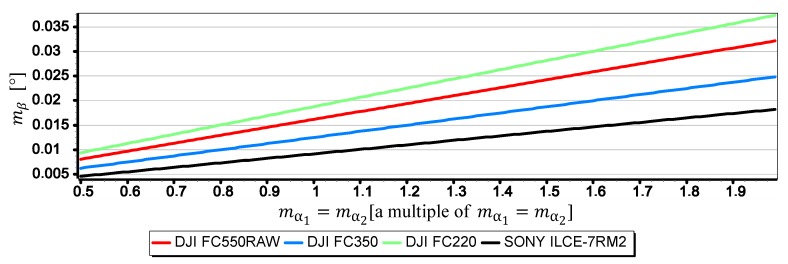
Mean error of measurement $m_{\beta }$ as a function of $m_{\alpha_{1}}=m_{\alpha_{2}}$ for β=10° (only the last two components of the Formula (3) were used in the calculations).

**Figure 26:**
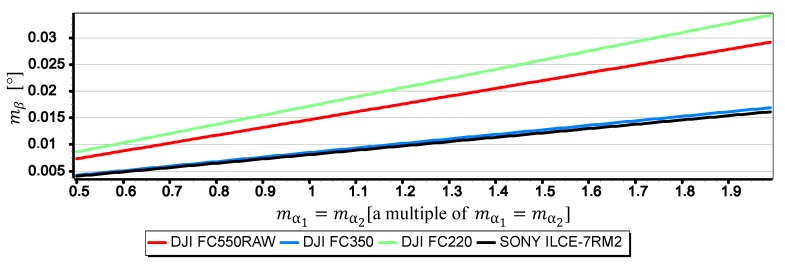
Mean error of measurement $m_{\beta }$ as a function of $m_{\alpha_{1}}=m_{\alpha_{2}}$ for β=15° (only the last two components of the Formula (3) were used in the calculations).

Figure 24, Figure 25 and Figure 26 present graphs of the mean error of measurement $m_{\beta }$ as a function of mean error of angles $m_{\alpha_{1}}$ i $m_{\alpha_{2}}$ measurements on CMOS matrix for the horizon slop β equals 5°, 10°, 15°. It was assumed that the values of $m_{\alpha_{1}}$ and $m_{\alpha_{2}}$ (calculated by Dependence (14)) are equal and correspond to their multiple in the range of $\left(0.5m_{\alpha_{1,2}},2m_{\alpha_{1,2}}\right)$. Thanks to this, as in the case of m_w, it was possible to carry out an analysis of the impact of $m_{\alpha_{1}}$ and $m_{\alpha_{2}}$ on $m_{\beta }$ using the comparative method for all the cameras simultaneously even though they have different focal lengths and matrix sizes.

**Figure 27:**
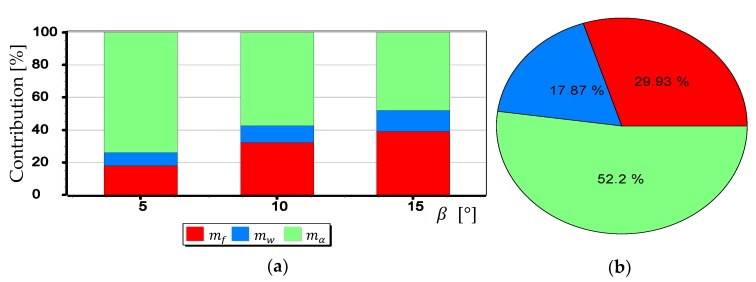
Percentage contribution for SONY ILCE-7RM2 camera: (**a**) Calculated for β equals 5°, 10° and 15°; (**b**) Calculated as a mean value.

**Figure 28:**
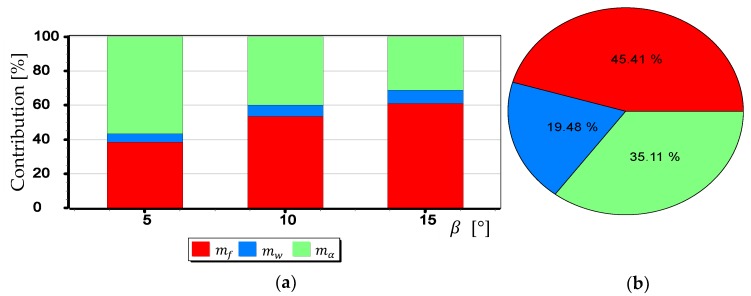
Percentage contribution for DIJ FC220 camera: (**a**) Calculated for β equals 5°, 10° and 15°; (**b**) Calculated as a mean value.

**Figure 29:**
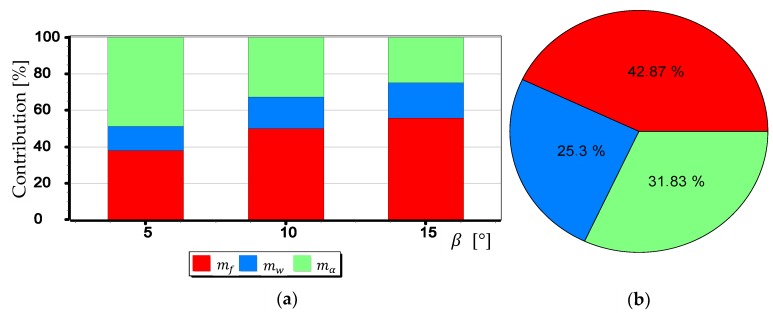
Percentage contribution for DIJ FC350 camera: (**a**) Calculated for β equals 5°, 10° and 15°; (**b**) Calculated as a mean value.

**Figure 30:**
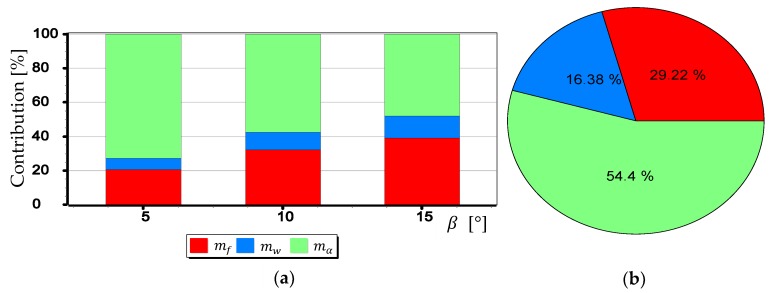
Percentage contribution for DIJ FC550RAW camera: (**a**) Calculated for β equals 5°, 10° and 15°; (**b**) Calculated as a mean value.

The graphs presented in Figure 24, Figure 25 and Figure 26 indicate that the influence of $m_{\alpha_{1}}$ and $m_{\alpha_{2}}$ on $m_{\beta }$ depends primarily on the size of the angle β being measured. Although in this case, contrary to the case of $m_{f}$, increasing the value of the angle *β* results in decreasing the value of $m_{\beta }$. It should also be noted that in the case of DJI FC350 and DJI FC550RAW cameras, for the doubled value $m_{\alpha_{1}}=m_{\alpha_{2}}$ and β=5°, the calculated value of $m_{\beta }$ reached as much as 0.035°. Figure 27, Figure 28, Figure 29 and Figure 30 present influance of $m_{f},m_{w},m_{\alpha_{1}},m_{\alpha_{2}}$ on $m_{\beta }$ as a percentage contribution.

Based on the graphs presented in Figure 27, Figure 28, Figure 29 and Figure 30, two generalized conclusions can be made:

- $m_{w}$ has the least effect on the value of $m_{\beta }$;
- for small β angles $m_{\alpha_{1}}$ i $m_{\alpha_{2}}$ have the greatest impact on the value of $m_{\beta }$, while for larger ones-$m_{f}$.

## 4. Conclusions

The method of camera evaluation presented in the paper can be considered as quite specific because it is based on the analysis of the optical system in combination with the way it is used to measure the angular position relative to the sea horizon. However, the proposed approach allows using the method to evaluate cameras mounted on USV or UAV taking the height of the sea wave and the height of the camera into account. It has been confirmed by the evaluation results of cameras mounted on UAV manufactured by DJI. The results led to the following general conclusions:

1. The image of the sea horizon can be curved already at camera heights h ≅ 10 m, and with the increase in height and the camera field of view, this curvature can increase. At a height of h=200 m and a horizontal field of view of the camera $FOV_{H}=120{}^{\circ}$, horizon sagitta $s_{r}$ can be as much as eight pixels (see Figure 6). Therefore, it is necessary to take this phenomenon into account in angular measurements made relative to the sea horizon using a UAV camera (especially those carried out to the entire length of the horizon line in the camera’s field of view).
2. For greater resolution of a single-pixel measurement of the horizon line, the maximum focal length should be used (see Figure 8, Figure 9 and Figure 10). Nevertheless, it should be remembered that the horizon is almost always distorted at the contact with the sea by rippling the water surface. Therefore, increasing the measurement resolution of a single-pixel to values smaller than the height of the sea waves will certainly not increase the resolution of the horizon slop measurement.
3. The measurement resolution of the horizon slope $r_{\beta }$ with a middle-class camera (of course, today class) can be at the level of 0.02° (see Table 2, the second part of the Formula (8)). However, it should be borne in mind that up to a given camera height limit, the resolution depends not only on the parameters of the optical system but also on the height of the sea waves (see Figure 15 and Table 3). In the case of the rated cameras for the wave height “L”=2 m, the threshold value of $h_{c_{\beta }/r_{\beta }}$ was as much as 21.7 m. Therefore, the measurement resolution using such ASV cameras should be calculated as the $c_{\beta }$ value, taking the wave height (using the first equation of Dependence (8)) into account. On the other hand, the switch to the resolution value $r_{\beta }$ can take place only after reaching the camera height h ≥ 21.7 m. It means, it rather will concern measurements made from UAV.
4. The mean errors $m_{\beta }$ of the horizon slope β measurements of the rated cameras were at the level of 0.02° and were similar to the resolution of measurement (see Figure 16). Its value decreases significantly with the increase of the horizon slope β (see Figure 19) and the increase of the focal length (see Figure 17 and Figure 18). Therefore, to measure the horizon slope with the smallest value of the mean error ($m_{\beta }=min$), the focal length should be set on a maximum and the camera should be rotated relative to the optical axis so that the horizon line would be along the diagonal of the matrix.
5. The mean error of measuring the focal length $m_{f}$ can have a significant impact on $m_{\beta }$. This applies especially to the use of light cameras with focal lengths of short length f<5 mm, when the ratio $m_{f}$ to f is relatively high (it reaches the value of the order of thousandths by a millimeter). Measurements of the tilt angle β with such cameras for $m_{f}=20$ μm (this is a high accuracy of focal length measurement) will be made with $m_{\beta }$ which is increased by a minimum of 0.03°.
6. $m_{\alpha_{1}}\;\mathrm{and}\;m_{\alpha_{2}}$ can also have a significant impact on $m_{\beta }$. This applies in particular to the use of larger cameras with focal lengths f>15 mm and matrices with sizes exceeding 4/3′′. The results of measurements with such cameras may be affected by $m_{\beta }$ error up to 70% dependent on $m_{\alpha_{1}}\;\mathrm{and}\;m_{\alpha_{2}}$. For small tilt angles β-below 5° the value of $m_{\beta }$ can increase by up to several hundredths of a degree.
7. The obtained values of the mean error of measurement $m_{\beta }$ of the horizon slope β for the tested cameras are slightly higher than INSs used on USV, but much smaller than INSs used on UAV [9,10,11,12]. Therefore, measuring the pitch and roll of bathymetric surveying vehicles using a camera can be considered reasonable. Although, it must be realized that there are other more important factors affecting the accuracy of optical measurement: lens distortion, diffraction limited, motion artifact and lowering of the visibility-omitted in the tests.

Taking the presented general and detailed conclusions into account (resulting from the assessment) the usability of cameras for determining the angular orientation of unmanned bathymetric surveying vehicles, based on the image of the sea horizon should be considered. Therefore, further research on this type of tilt compensator is justified and advisable. The activity could be directed at:

- adaptation of recording and image processing equipment to marine conditions (including various external lighting and lighting from the water table),
- appropriate selection of image processing methods with emphasis on changes in visibility (caused by fog, rainfall or snowfall),
- conducting several verification tests of the compensator prototype in a real environment (to find, remove or minimize the so-called critical functions of technology),
- in the longer term, the methods for its use will be developing, including using for stabilization of UAV or USV spatial orientation, relative positioning (e.g., in relation to floating navigational marks or seabed), or for precise determination of the direction and distance to the floating surface object or underwater object (optically, as well as supporting the work of navigation devices such as: sonar, radar or LiDAR).

## Figures and Tables

**Table 1:** Technical parameters of evaluated UAV cameras.

Type of Camera and Gimbal		Optical Parameters
Camera: DJI FC350 Gimbal: Zenmuse X3	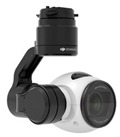	CMOS size: 1/2.3′′ Image size: 4000 × 3000 pixels Focal length: 4–14 mm https://store.dji.com/product/zenmuse-x3-gimbal-camera (accessed on 16 July 2019)
Camera: DJI FC220 Gimbal: DJI Mavic Pro	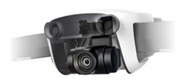	CMOS size: 1/2.3′′ Image size: 4000 × 3000 pixels Focal length: 5 mm https://www.dji.com/pl/mavic?site=brandsite&from=landing_page (accessed on 16 July 2019)
Camera: DJI FC550RAW Gimbal: Zenmuse X5S	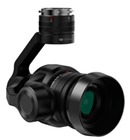	CMOS size: 4/3′′ Image size: 4608 × 3456 pixels Focal length: 15 mm https://store.dji.com/product/zenmuse-x5s?site=brandsite&from=buy_now_bar (accessed on 16 July 2019)
Camera: SONY ILCE-7RM2 Lens: SAMYANG 35 mm f/2.8 FE Gimbal: Zenmuse Z15-A7	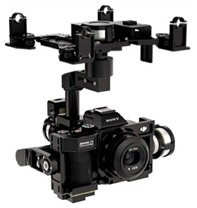	CMOS size: matrix full-frame (35 mm) Image size: 7952 × 5304 pixels Focal length: 35 mm https://www.sony.pl/electronics/aparaty-z-wymiennymi-obiektywami/ilce-7rm2 (accessed on 16 July 2019) http://www.samyang-foto.pl/pl/obiektyw-z-af/af-35mm-f28-fe1 (accessed on 16 July 2019) https://www.dji.com/pl/zenmuse-z15-a7 (accessed on 16 July 2019)

**Table 2:** Horizon slope measurement resolution $r_{\beta }$ and horizontal field of view $FOV_{H}$ calculated for cameras: DJI FC350, DJI FC220, DJI FC550RAW and SONY ILCE-7RM2.

Parameter	DJI FC220	DJI FC350 *f* = 4 mm/*f* = 14 mm	DJI FC550RAW	SONY ILCE-7RM2
$r_{\beta }$	0.0168°	0.0181°/0.0147°	0.0144°	0.0081°
$FOV_{H}$	63.27°	75.19°/24.81°	59.94°	53.91°

**Table 3:** Threshold values of height $h_{c_{\beta }/r_{\beta }}$ above which $r_{\beta }$ should be used instead of $c_{\beta }$ ($c_{\beta }≅r_{\beta }$).

Parameter	DJI FC220	DJI FC350 *f* = 14 mm	DJI FC550RAW	SONY ILCE-7RM2
$h_{c_{\beta }/r_{\beta }}$ for (“L”=0.5 m)	0.2 m	1.4 m	0.3 m	0.3 m
$h_{c_{\beta }/r_{\beta }}$ for (“L”=1.0 m)	0.7 m	5.4 m	1.1 m	4.0 m
$h_{c_{\beta }/r_{\beta }}$ for (“L”=1.5 m)	1.6 m	12.2 m	2.4 m	9.0 m
$h_{c_{\beta }/r_{\beta }}$ for (“L”=2.0 m)	2.8 m	21.7 m	4.2 m	16.0 m

**Table 4:** Mean error of measurement $m_{\beta }$ calculated for β=5° i β=10° assumig that $m_{f}=10$ μm (only the first component of the Formula (3) was used).

Parameter	DJI FC220	DJI FC350 *f* = 14 mm	DJI FC550RAW	SONY ILCE-7RM2
$m_{\beta }$ for (β=10°)	0.0250°	0.01942°	0.0094°	0.0044°
$m_{\beta }$ for (β=15°)	0.0365°	0.01905°	0.0191°	0.0057°
